# Establishing disease-specific thresholds for automated hematopoietic progenitor cell counting in hematological malignancies

**DOI:** 10.1371/journal.pone.0333196

**Published:** 2025-10-08

**Authors:** Orathai Taka, Anchalee Thedsawad, Weerapat Owattanapanich

**Affiliations:** Division of Hematology, Department of Medicine, Faculty of Medicine Siriraj Hospital, Mahidol University, Bangkok Noi, Bangkok, Thailand; European Institute of Oncology, ITALY

## Abstract

**Background:**

Flow cytometry remains the gold-standard method for enumerating CD34 + cells during peripheral blood stem cell (PBSC) transplantation, but it is resource- and time-intensive. This study aimed to assess the correlation between hematopoietic progenitor cell (HPC) counts measured by a Sysmex XN-series analyzer and CD34 + cell counts obtained by flow cytometry in pre-apheresis peripheral blood and apheresis products, and to establish HPC cutoffs for predicting adequate PBSC yields.

**Methods:**

We analyzed 334 samples (167 pre-apheresis peripheral blood, 167 PBSC apheresis products) collected from patients and healthy donors. HPC and CD34 + cell counts were measured in all samples. Receiver operating characteristic (ROC) curve analysis was performed to determine HPC cutoff values predictive of adequate PBSC harvest.

**Results:**

Intraclass correlation coefficients (ICCs) indicated strong HPC‒CD34 + concordance in pre-apheresis peripheral blood (ICC = 0.896, *p* < 0.001) and apheresis products (ICC = 0.958, *p* < 0.001). Median HPC counts were significantly higher than CD34 + cell counts overall (16 vs. 14.6/µL, *p* < 0.001; 1,040 vs. 804.73/µL, *p* < 0.001). The same trend was observed in multiple myeloma (50 vs. 23.92/µL, *p* < 0.001; 1,290 vs. 1,132.85/µL, *p* = 0.002) and lymphoma (7 vs. 2.48/µL, *p* = 0.011; 615 vs. 475.12/µL, *p* = 0.004). In healthy donors, median HPC counts in apheresis products also exceeded CD34 + cell counts (1,580 vs. 1,430.45/µL, *p* = 0.005). The HPC cutoffs predicting peripheral blood CD34+ > 20/µL and a CD34 + cell yield of ≥ 2 × 10^6^ cells/kg were 21/µL overall (sensitivity 91.5%, specificity 90.6%), 27/µL for multiple myeloma (87.2%, 81.3%), and 20/µL for lymphoma (83.3%, 95.2%).

**Conclusions:**

This study establishes the first disease-specific HPC cutoff values targeting the clinically critical 2 × 10^6^ cells/kg threshold: 27 cells/μL for multiple myeloma and 20 cells/μL for lymphoma. This precision medicine approach advances beyond universal cutoffs, enabling optimized collection timing and potentially reducing healthcare costs.

## Introduction

Hematopoietic stem cell transplantation has long been an effective treatment for certain hematolymphoid malignancies, solid tumors, and inborn errors of immunity and metabolism [1]. Data from a 2006 *Journal of the American Medical Association* report indicated that the Asia-Pacific region performed approximately 10%‒20% of all global hematopoietic stem cell transplantation procedures that year [2].

Hematopoietic stem cells can be harvested from bone marrow or peripheral blood via apheresis. Peripheral blood stem cell (PBSC) collection typically requires mobilization of bone marrow stem cells with chemotherapy and hematopoietic growth factors [3]. Compared to bone marrow harvest, PBSC collection avoids surgical procedures and general anesthesia, and it accelerates platelet and neutrophil engraftment [4].

Successful PBSC transplantation requires an adequate dose of hematopoietic progenitor cells (HPCs) to ensure rapid and sustained hematologic recovery. The timing of apheresis is usually guided by measuring CD34 + cells through conventional flow cytometry, following International Society of Hematotherapy and Graft Engineering guidelines [5]. However, this procedure is labor-intensive, costly, and demands well-trained personnel.

Newer-generation automated hematology analyzers are increasingly being adopted in clinical laboratories worldwide. Many feature advanced parameters to detect blood elements such as immature platelets and granulocytes, which aid in disease evaluation and monitoring. The Sysmex XN-series analyzer (Sysmex Corporation, Kobe, Japan) is a 5-part differential hematology analyzer [6] that incorporates multiple specialized measurement channels, including the White Precursor Cell (WPC) channel for detecting and enumerating HPCs. The XN-HPC parameter, which utilizes the WPC channel, is available on the fully automated Sysmex XN-20, and is used to assess the yield of HPCs [7]. Its automated HPC counting relies on fluorescence flow cytometry that considers membrane permeability, cell size, and internal complexity.

Previous studies [8,9] have demonstrated a strong correlation between XN-derived HPC enumeration (XN-HPC) and conventional flow cytometry-based CD34 + cell counts. Moreover, the Sysmex XN-series uses only 190 μL of peripheral blood or apheresis product, offering a rapid, non-labor-intensive turnaround in about 120 seconds. This efficiency could streamline PBSC apheresis management, reducing overall costs. However, existing studies have not established disease-specific cutoff values directly targeting the clinically critical 2 × 10^6^ cells/kg threshold, representing a significant gap in precision medicine approaches to PBSC collection.

In this study, we assessed the correlation between Sysmex XN-HPC and flow cytometry-derived CD34 + cell counts in peripheral blood and apheresis products. We also established cutoff values of XN-HPC counts in pre-apheresis peripheral blood to predict adequate HPC yield in the harvested product.

## Materials and methods

### Power analysis and sample size justification

We determined the study’s sample size through a power analysis designed to detect meaningful differences with sufficient statistical power. We used an estimated sensitivity of 0.735 from a previous study by Kim et al. [10]. A standard normal value (Z) of 1.96 at 95% confidence and a 0.10 margin of error were also applied. The formula to estimate the required sample size was based on diagnostic test sensitivity. Considering these parameters, plus potential dropout and data variability, we initially calculated a minimum requirement of approximately 75 samples to achieve a 10% margin of error for sensitivity at 95% confidence. However, to compare the prevalence of 45% reported by Kim et al. [10], we adjusted the total to 167 samples. Consequently, we included 167 pre-apheresis peripheral blood samples and 167 PBSC apheresis samples in this study.

### Blood samples

**Fig 1:**
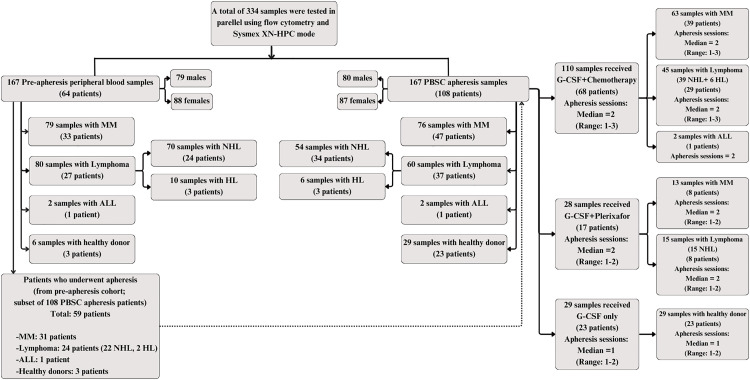
Flow diagram of sample selection and classification. The diagram outlines the distribution of pre-apheresis peripheral blood and peripheral blood stem cell apheresis samples, categorized by diagnosis and donor type, and includes the median number of apheresis sessions according to mobilization strategies. **Abbreviations:** ALL, acute lymphoblastic leukemia; G-CSF, granulocyte colony-stimulating factor; HL, Hodgkin lymphoma; MM, multiple myeloma; NHL, Non-Hodgkin lymphoma; PBSC, peripheral blood stem cell; Sysmex XN-HPC, Sysmex XN-derived hematopoietic progenitor cell enumeration.

We analyzed 334 samples, comprising 167 pre-apheresis peripheral blood samples and 167 PBSC apheresis samples. The pre-apheresis peripheral blood samples were obtained from 3 healthy donors and 61 PBSC transplant recipients (33 with multiple myeloma [MM], 27 with lymphoma, and 1 with acute lymphoblastic leukemia). Among these individuals, 31 MM patients, 24 lymphoma patients, 1 acute lymphoblastic leukemia patient, and 3 healthy donors underwent PBSC apheresis, comprising the PBSC apheresis cohort. All patients in the PBSC apheresis cohort had matched pre-apheresis CD34 + data and were therefore also included in the pre-apheresis cohort (Fig 1). The PBSC apheresis samples were obtained from 23 healthy donors and 85 PBSC transplant recipients (47 with MM, 37 with lymphoma, and 1 with acute lymphoblastic leukemia), including those previously described.

These were leftover blood samples from autologous hematopoietic stem cell transplantation in patients with hematologic malignancies and from allogeneic donors. All samples were collected for routine flow cytometry‒based CD34 + enumeration at the Division of Hematology, Department of Medicine, Faculty of Medicine Siriraj Hospital, Mahidol University, Bangkok, Thailand. Blood samples were accessed for research purposes from 01/03/2023–30/04/2024.

**Fig 2:**
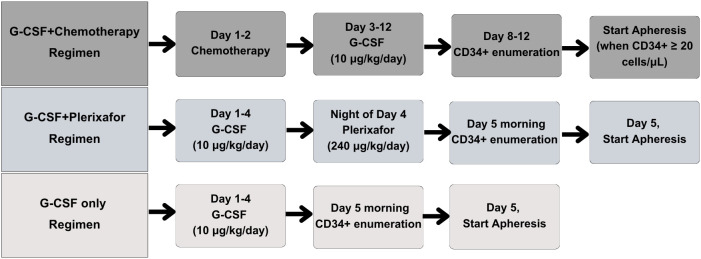
Flowchart summarizing mobilization strategies, including dosing of granulocyte colony-stimulating factor (G-CSF), plerixafor, chemotherapy, and timing of CD34 + cell count monitoring.

Pre-apheresis peripheral blood samples were collected in EDTA tubes, while PBSC apheresis products were obtained directly from the apheresis machine. Healthy donors were mobilized with granulocyte colony-stimulating factor (G-CSF) at a dose of 10 μg/kg/day. Patients with MM, lymphoma, or acute lymphoblastic leukemia received either chemotherapy followed by G-CSF at a dose typically ranging from 5 to 10 μg/kg/day, or G-CSF plus plerixafor. Peripheral blood CD34 + cell counts were monitored beginning on day 5 after the first administration of G-CSF, or between days 8 and 12 after chemotherapy-based mobilization. For donors and patients mobilized with G-CSF plus plerixafor, the pre-apheresis peripheral blood CD34 + value refers specifically to the day 5 morning sample, collected immediately prior to the first apheresis procedure. The mobilization regimens, including the timing and dosing of chemotherapy, G-CSF, plerixafor, and CD34 ⁺ monitoring, are shown in Fig 2. Stem cell apheresis for all donors and patients was performed using the Spectra Optia apheresis system (Terumo Corporation, Tokyo, Japan).

All samples were analyzed for both CD34 + enumeration by flow cytometry and XN-HPC by the Sysmex XN-20 automated hematology analyzer at the same time, within 2 hours of collection, during the same testing session. This study was approved by the Siriraj Institutional Review Board (SIRB), Faculty of Medicine Siriraj Hospital, Mahidol University (reference: Si 822/2022) and conducted in accordance with the Declaration of Helsinki (as revised in 2013). The IRB approved a waiver of informed consent due to the use of anonymized leftover samples collected after routine clinical laboratory analysis. No identifiable personal data were included.

### Enumeration of CD34 + cell count by flow cytometry and Sysmex XN-HPC in pre-apheresis PB and PBSC apheresis groups

During mobilization, subjects were sampled daily until their peripheral blood CD34 + cell count exceeded 20 cells/µL, at which point the apheresis process began. The final apheresis product was measured once to evaluate stem cell yield. For flow cytometry testing, samples from the PBSC apheresis group were diluted 1:10 with 1 × PBS and counted by an automatic blood cell counter, ensuring the post-dilution white blood cell count remained below 40,000 cells/µL.

We used the BD Stem Cell Enumeration kit (BD Biosciences, San Jose, CA, USA) for staining CD45/CD34 surface antigens with a lyse/no-wash protocol. Briefly, 20 µL of stem cell reagent and 20 µL of 7-AAD were added to a BD Trucount tube (BD Biosciences), followed by 100 µL of EDTA blood (from pre-apheresis peripheral blood) or 100 µL of the 1:10 diluted apheresis sample. Samples were incubated for 20 minutes at room temperature in the dark. To improve accuracy, we used reverse pipetting when adding samples to the antibody mix. After incubation, 2 mL of ammonium chloride was added to lyse red blood cells, and tubes were gently vortexed before a further 10-minute dark incubation. Samples were then analyzed on a BD FACSVerse or BD LSR-Fortessa Flow Cytometer (BD Biosciences) within 1 hour. Results were reported as both absolute CD34 + cells/µL and percentage of CD34 + cells.

XN-HPC measurements were performed using the Sysmex XN-20, a fully automated hematology analyzer equipped with the WPC flow cytometry channel. HPCs are differentiated in the WPC by forward-scattered, side-scattered, and side-fluorescent light signals [11,12]. Fluorescent intensity depends on cell membrane permeability to the WPC reagent, which perforates lipid-rich membrane regions [13]. Stem cells resist this permeabilization because of their low membrane lipid content, thereby exhibiting high forward-scattered, low side-scattered, and low side-fluorescent signals. This profile distinguishes HPCs from other cell populations. Analysis requires 190 µL of EDTA blood or PBSC apheresis product. For apheresis products, we diluted samples 1:10 with 1 × PBS to avoid reagent depletion from high stem cell concentrations [14]. The XN-HPC count is reported as both absolute HPCs/µL and a percentage of total white blood cells [8,11]. Quality control was performed daily using XN-Chex control blood (Sysmex Corp.), a three-level control material.

Peripheral blood CD34 + cell counts were reported as cells/µL, whereas CD34 + yields from the apheresis collection were expressed as cells/kg of recipient body weight.

### Statistical analysis

Data were analyzed using IBM SPSS Statistics version 22 (IBM Corp., Armonk, NY, USA) and MedCalc version 23.0.8 (MedCalc Software Ltd., Ostend, Belgium). Normal distribution was tested with the Kolmogorov‒Smirnov or Shapiro‒Wilk test. The Wilcoxon signed-rank test compared absolute HPC and absolute CD34 + cell counts between Sysmex XN-HPC and flow cytometry. The intraclass correlation coefficient (ICC) assessed consistency between the two methods. Passing‒Bablok regression and Spearman’s rank correlation coefficient evaluated linear relationships, where the regression intercept indicates constant mean bias and the slope indicates proportional bias. Bland‒Altman plots were used to assess agreement by plotting the difference on the y-axis against the mean on the x-axis.

Receiver operating characteristic analysis determined the optimal Sysmex XN-HPC cutoff. Flow cytometry’s peripheral blood CD34 + cutoff of 20 cells/μL [8] reliably predicts a yield of 2 × 10^6^ cells/kg. This clinically relevant threshold was used to define corresponding XN-HPC cutoffs with sensitivity, specificity, positive predictive value (PPV), negative predictive value (NPV), and area under the curve (AUC). A *p*-value < 0.05 was considered statistically significant.

## Results

### Sample characteristics and mobilization regimens

A total of 334 samples were analyzed in parallel via flow cytometry and Sysmex XN-HPC mode, comprising 167 pre-apheresis peripheral blood samples and 167 PBSC apheresis samples. Among the pre-apheresis samples, 79 were from patients with MM, 80 from patients with lymphoma, 2 from individuals with acute lymphoblastic leukemia, and 6 from healthy donors. In the PBSC apheresis group, 76 samples were from MM patients, 60 from lymphoma patients, 2 from individuals with acute lymphoblastic leukemia, and 29 from healthy donors. Non-Hodgkin lymphoma was the most frequent subtype among lymphoma cases, followed by Hodgkin lymphoma. The median age of participants ranged from 13 to 79 years, and both groups were predominantly female (Fig 1). The number of pre-apheresis samples exceeded the number of patients because, during stem cell mobilization, peripheral blood samples were collected daily to monitor CD34 + cell counts until the threshold for initiating apheresis was reached. As a result, a single patient could contribute multiple pre-apheresis samples. Additionally, some patients did not reach the CD34 + threshold, resulting in cancellation of the apheresis procedure and contributing only pre-apheresis data. Furthermore, it is common in clinical practice to perform additional apheresis sessions when the initial collection does not reach the target dose, resulting in more post-apheresis samples than patients.

### Correlation of absolute HPC and CD34 + cell counts in the pre-apheresis peripheral blood group

We compared absolute HPC and absolute CD34 + cell counts in 167 pre-apheresis peripheral blood samples (Table 1). Overall, the median absolute HPC count was significantly higher than the absolute CD34 + cell count (16 cells/µL, interquartile range [IQR] 6‒54 vs. 14.6 cells/µL, IQR 1.4‒46.2; *p *< 0.01). The ICC was 0.896 (*p *< 0.001), indicating good agreement between the two methods.

**Table 1:** Demographic and clinical characteristics, cell counts, and method comparison metrics between absolute hematopoietic progenitor cell and CD34 + cell counts in pre-apheresis peripheral blood and peripheral blood stem cell apheresis samples.

Groups		Pre-apheresis PB					PBSC apheresis				
		All	Multiple myeloma	Lymphoma (NHL + HL)	Acute lymphoblastic leukemia	Healthy donors	All	Multiple myeloma	Lymphoma (NHL + HL)	Acute lymphoblastic leukemia	Healthy donors
**No. of patients**		64	33	27 (24 + 3)	1	3	108	47	37 (34 + 3)	1	23
**No. of samples**		167	79	80 (70 + 10)	2	6	167	76	60 (54 + 6)	2	29
**Age (years), median (range)**		54 (18-66)	59 (43-65)	49 (18-66)	25	43 (31-47)	52 (13-79)	59 (43-67)	50 (20-79)	25	36 (13-61)
**Sex, M:F, n**		79:88	36:43	40:40	1:0	1:5	80:87	36:40	29:31	1:0	13:16
**Sysmex XN-HPC**	**Absolute HPC count (cells/μL), median (IQR)**	16 (6-54)	50 (11-110)	7 (4-17)	23 (12–23)^a^	37 (21-65)	1,040 (560−1,810)	1,290 (651−3,168)	615 (330−1,018)	855 (540–855)^a^	1,580 (1,060−2,288)
	**CD34 + cell yield,** **10**^**6**^ **cells/kg, median (IQR)**						4.72 (2.26-9.18)	5.73 (3.26-11.70)	2.84 (1.49-4.79)	3.37 (2.05–3.37)^a^	9.18 (4.79-12.69)
**Flow cytometry**	**Absolute CD34+** **(cells/μL), median (IQR)**	14.63 (1.45-46.23)	23.92 (11.71-88.57)	2.48 (0.53-17.37)	30.96 (18.07–30.96)^a^	34.47 (22.12-48.01)	804.73 (392.45−1,709.74)	1,132.85 (437.13−2,175.18)	475.12 (222.93-907.94)	1,093.66 (632.88−1,093.66)^a^	1,430.45 (855.50−1,961.35)
	**CD34 + cell yield,** **10**^**6**^ **cells/kg, median (IQR)**						4.01 (1.85-8.27)	4.91 (2.07-10.81)	2.15 (0.98-4.61)	4.31 (2.40–4.31)^a^	8.10 (4.40-10.18)
**ICC (95% CI)**		0.896 (0.861-0.922)	0.884 (0.824-0.924)	0.871 (0.805-0.915)	0.979 (0.742-1.000)	0.924 (0.561-0.989)	0.958 (0.943-0.969)	0.956 (0.932-0.972)	0.964 (0.940-0.978)	0.932 (0.916-1.000)	0.899 (0.797-0.951)
**Bland‒Altman difference plot**	**Mean bias (95% CI)**	9.0 (3.3-14.6)	17.6 (6.1-29.1)	1.0 (–1.2 to 3.3)	N/A	7.4 (–4.2 to 18.9)	138.9 (55.6-222.3)	148.7 (–8.70 to 306.12)	75.7 (–13.22 to 164.61)	N/A	270.3 (98.03-442.50)
	**95% limits of agreement** **(mean bias ± 1.96 SD)**	–63.2 to 81.2	–82.8 to 118.0	−18.9-21.0	N/A	–14.3 to 29.0	–930.2 to 1,208.1	–1,201.5 to 1,498.9	–598.9 to 750.3	N/A	–617.2 to 1,157.7
**Passing‒Bablok regression**	**Slope** **(95% CI)**	1.068 (0.990-1.129)	1.100 (1.028-1.198)	0.866 (0.746-1.047)	N/A	1.615 (0.848-3.404)	1.014 (0.956-1.078)	0.990 (0.922-1.0598)	0.979 (0.850-1.142)	N/A	1.124 (0.853-1.400)
	**Intercept** **(95% CI)**	1.989 (1.671-2.663)	1.725 (–0.408 to 3.356)	2.562 (1.885-3.090)	N/A	–19.559 (–76.328 to 6.706)	128.704 (65.084-164.046)	132.440 (57.727-210.936)	96.699 (54.571-182.192)	N/A	98.319 (–286.032 to 390.636)

Abbreviations: CI, confidence interval; HL, Hodgkin lymphoma; HPC, hematopoietic stem cell; IQR, interquartile range; N/A, not applicable; NHL, non-Hodgkin lymphoma; PB, peripheral blood; PBSC, peripheral blood stem cell; Sysmex XN-HPC, Sysmex XN-derived hematopoietic progenitor cell enumeration.

^a^Q3 not applicable for n = 2.

In subgroup analyses by diagnosis, the median absolute HPC count was significantly higher than the absolute CD34 + cell count in MM (50 cells/µL, IQR 11‒110 vs. 23.92 cells/µL, IQR 11.7‒88.57; *p *< 0.001) and lymphoma (7 cells/µL, IQR 4‒17 vs. 2.48 cells/µL, IQR 0.53‒17.37; *p *= 0.011). However, no significant difference emerged in acute lymphoblastic leukemia (23 cells/µL, IQR 12‒23 vs. 30.96 cells/µL, IQR 18.07‒30.96; *p *= 0.180). In healthy donors, due to the limited number of pre-apheresis peripheral blood samples (n = 6), no significant difference was observed between the absolute HPC and CD34 + cell counts (37 vs. 34.47/µL; *p* = 0.116). This small sample size reflects clinical practice, in which pre-apheresis testing is often omitted in healthy donors because successful stem cell collection is generally expected. The ICC showed good agreement for MM (0.884) and lymphoma (0.871), and excellent agreement for healthy donors (0.924).

**Fig 3:**
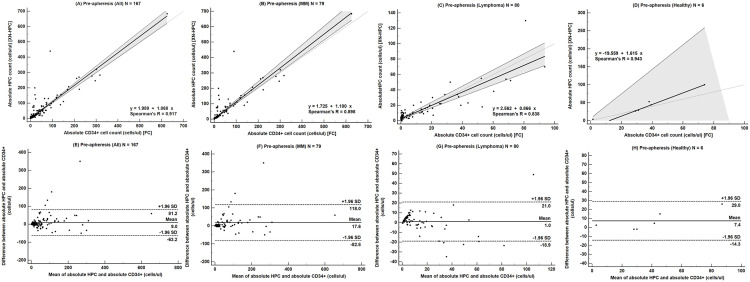
Comparison of absolute hematopoietic progenitor cell (HPC) counts and CD34 + cell counts in pre-apheresis peripheral blood samples using Passing‒Bablok regression and Bland‒Altman analyses. Panels A‒D show Passing‒Bablok regression analyses for (A) all patients, (B) patients with multiple myeloma, (C) patients with lymphoma, and (D) healthy donors. The thick solid line represents the regression line, with the shaded area indicating the 95% confidence interval (CI) of the slope. The thin solid line represents the line of identity (y = x). Panels E–H show corresponding Bland‒Altman plots for (E) all patients, (F) patients with multiple myeloma, (G) patients with lymphoma, and (H) healthy donors. The solid line indicates the mean difference (bias), and the dot-dashed lines represent the upper and lower limits of agreement. **Abbreviations:** HPC, hematopoietic progenitor cell; MM, multiple myeloma; XN-HPC, Sysmex XN-derived hematopoietic progenitor cell enumeration.

Passing‒Bablok regression analysis of all samples, as well as MM, lymphoma, and healthy donor subgroups, showed high correlation between Sysmex XN-HPC and flow cytometry (r = 0.917, *p *< 0.001). The slopes were 1.068, 1.100, 0.866, and 1.615, and the intercepts were 1.989, 1.725, 2.562, and ‒19.559, respectively (Fig 3A–D). Bland‒Altman plots demonstrated a mean absolute bias of 9.0 cells/µL (95% limits of agreement: ‒63.2 to 81.2) for the overall cohort, 17.6 cells/µL (‒82.8 to 118.0) in MM, 1.0 cell/µL (‒18.9 to 21.0) in lymphoma, and 7.4 cells/µL (‒14.3 to 29.0) in healthy donors (Fig 3E–H).

### Correlation of absolute HPC and CD34 + cell counts in the PBSC apheresis group

A total of 167 samples were analyzed in the PBSC apheresis group (Table 1). The median absolute HPC count was significantly higher than the absolute CD34 + cell count in all samples. The HPC median was 1,040 cells/µL (IQR 560‒1,810) compared with 804.73 cells/µL (IQR 392.45‒1,709.74; *p* < 0.001). This trend was also observed in MM, where the HPC median was 1,290 cells/µL (IQR 651‒3,168) vs. 1,132.85 cells/µL (IQR 437.13‒2,175.18; *p *= 0.002). A similar result was found in lymphoma (615 vs. 475.12 cells/µL; *p *= 0.004) and in healthy donors (1,580 vs. 1,430.45 cells/µL; *p *= 0.005). ICCs showed excellent agreement between Sysmex XN-HPC and flow cytometry (0.958 overall, 0.956 in MM, 0.964 in lymphoma, 0.899 in donors). Spearman’s correlation was also very strong (r = 0.932, *p *< 0.001).

**Fig 4:**
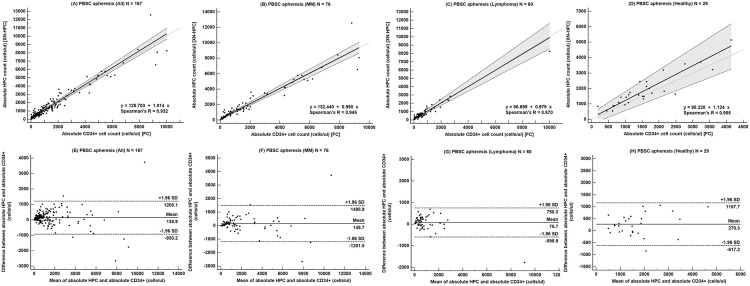
Comparison of absolute hematopoietic progenitor cell (HPC) counts and CD34 + cell counts in peripheral blood stem cell (PBSC) apheresis samples using Passing‒Bablok regression and Bland‒Altman analyses. Panels A‒D show Passing‒Bablok regression analyses for (A) all patients, (B) patients with multiple myeloma, (C) patients with lymphoma, and (D) healthy donors. The thick solid line represents the regression line, with the shaded area indicating the 95% confidence interval (CI) of the slope. The thin solid line represents the line of identity (y = x). Panels E‒H show corresponding Bland‒Altman plots for (E) all patients, (F) patients with multiple myeloma, (G) patients with lymphoma, and (H) healthy donors. The solid line indicates the mean difference (bias), and the dot-dashed lines represent the upper and lower limits of agreement. **Abbreviations:** HPC, hematopoietic progenitor cell; MM, multiple myeloma; PBSC, peripheral blood stem cell; XN-HPC, Sysmex XN-derived hematopoietic progenitor cell enumeration.

Passing‒Bablok regression revealed a significant constant bias overall (slope = 1.014, intercept = 128.704; Fig 4A). The Bland‒Altman plot confirmed this bias, with a mean difference of 138.9 cells/µL (95% CI 55.6‒222.3; Fig 4E). Subgroup analyses demonstrated significant constant bias in MM and lymphoma using Passing‒Bablok (slope = 0.990, intercept = 132.440; slope = 0.979, intercept = 96.699) (Fig 4B, C). However, Bland‒Altman plots did not confirm this bias (mean difference: 148.7 cells/µL, 95% limits –1,201.5 to 1,498.9 for MM; 75.7 cells/µL, –598.9 to 750.3 for lymphoma) (Fig 4F, G). Among healthy donors, the mean difference was also significant (270.3 cells/µL, –617.2 to 1,157.7) (Fig 4H). Using an absolute CD34 + threshold of > 2 × 10^6^ cells/kg, the CD34 + cell yield (cells/kg) calculated from HPC counts exceeded that derived from CD34 + cell counts in all groups except acute lymphoblastic leukemia. This discrepancy may be related to the small sample size in acute lymphoblastic leukemia.

### Absolute HPC count cutoff for the pre-apheresis PB group

Out of 167 pre-apheresis peripheral blood samples, 71 (42.5%) had an absolute CD34 + cell count ≥ 20 cells/μL. These samples also reached a CD34 + cell yield > 2 × 10^6^ cells/kg in the apheresis product and were thus deemed positive. The overall optimal cutoff of absolute HPC count for predicting an absolute CD34 + cell count ≥ 20 cells/μL was 21 cells/μL. This cutoff yielded an AUC of 0.961 (95% CI 0.934‒0.989), with 91.5% sensitivity, 90.6% specificity, 93.5% NPV, and 87.8% PPV.

Subgroup analysis identified an optimal HPC cutoff of 27 cells/μL in MM and 20 cells/μL in lymphoma. In MM, the AUC was 0.940 (95% CI 0.891‒0.990), with 87.2% sensitivity, 81.3% specificity, 81.3% NPV, and 87.2% PPV. In lymphoma, the AUC was 0.967 (95% CI 0.922‒1.000), with 83.3% sensitivity, 95.2% specificity, 95.2% NPV, and 83.3% PPV (Table 2). In MM patients, the absolute HPC cutoff was higher than the CD34 + threshold for predicting adequate apheresis.

**Table 2:** Receiver operating characteristic analysis of absolute hematopoietic progenitor cell count in pre-apheresis peripheral blood for predicting adequate CD34 + cell yield.

Absolute HPC count	Total samples	AUC (95% CI)	Cutoff^a^ (cells/µL)	Sensitivity (%)	Specificity (%)	NPV (%)	PPV (%)
**All**	167	0.961 (0.934-0.989)	21	91.5	90.6	93.5	87.8
**Multiple myeloma**	79	0.940 (0.891-0.990)	27	87.2	81.3	81.3	87.2
**Lymphoma (NHL + HL)**	80	0.967 (0.922-1.000)	20	83.3	95.2	95.2	83.3

Abbreviations: AUC, area under the curve; CI, confidence interval; HL, Hodgkin lymphoma; HPC, hematopoietic stem cell; NHL, non-Hodgkin lymphoma; NPV, negative predictive value; PPV, positive predictive value.

^a^Cutoff value of absolute HPC count on peripheral blood for a cell count of > 2 × 10^6^ CD34 + cells/kg in harvest product.

### Cell counts in mobilized PBSC collections and CD34 + cell yields

Of the 167 PBSC apheresis samples, 110 were mobilized with G-CSF plus chemotherapy, 28 with G-CSF plus plerixafor, and 29 with G-CSF alone (Fig 1). Across these three mobilization regimens, the median CD34 + cell yields (per kg) calculated from the absolute HPC count exceeded those based on the absolute CD34 + cell count in all groups except acute lymphoblastic leukemia. Thus, Sysmex XN-HPC consistently showed higher yield estimates than flow cytometry.

We observed a very strong correlation (r = 0.946‒0.963, *p *< 0.01) between absolute HPC and CD34 + cell yields, and between absolute CD34 + cell count and CD34 + cell yields (r = 0.955‒0.978, *p *< 0.01). Across all samples, these correlations were 0.963 and 0.972, respectively (Table 3). No significant differences emerged in patients mobilized with plerixafor (r = 0.953, r = 0.965) versus those mobilized without plerixafor (r = 0.963, r = 0.975). Similar findings were observed by diagnosis: in MM, the correlation coefficients were 0.955 and 0.972, and in lymphoma, 0.955 and 0.968. The overall correlations between absolute HPC and CD34 + cell counts in those mobilized with G-CSF plus chemotherapy, G-CSF plus plerixafor, or G-CSF alone were 0.928, 0.912, and 0.905, respectively (*p *< 0.001). MM patients attained higher CD34 + yields with G-CSF plus chemotherapy, whereas lymphoma patients showed higher yields with G-CSF plus plerixafor (Table 3).

**Table 3:** Comparison of hematopoietic progenitor and CD34 + cell counts and yields in peripheral blood stem cell apheresis products based on mobilization regimen.

Method	Total samples	Sysmex XN-HPC			Flow cytometry			Absolute HPC vs. absolute CD34 + cell count	
		Absolute HPC count (cells/μL) median (IQR)	CD34 + cell yield, 10^6^ cells/kg median (IQR)	Spearman r	Absolute CD34+ (cells/μL) median (IQR)	CD34 + cell yield, 10^6^ cells/kg median (IQR)	Spearman r	*p*-value^b^	Spearman r
**PBSC apheresis**									
**All regimens**									
**All**	167	1,040 (560−1,810)	4.72 (2.26-9.18)	0.963	804.73 (392.45−1,709.74)	4.01 (1.85-8.27)	0.972	<0.001	0.932
**Multiple myeloma**	76	1,290 (651−3,167)	5.74 (3.26-11.70)	0.955	1,132.85 (437.13−2,175.18)	4.92 (2.07-10.81)	0.972	0.002	0.945
**Lymphoma**	60	615 (330−1,017)	2.84 (1.49-4.79)	0.955	475.12 (222.93-907.94)	2.15 (0.98-4.61)	0.968	0.004	0.870
**G-CSF + chemotherapy**									
**All**	110	910 (480−1,783.75)	4.46 (2.05-8.40)	0.963	700.90 (331.22−1,619.26)	3.49 (1.65-7.53)	0.975	0.001	0.928
**Multiple myeloma**	63	1,290 (640−3,400)	6.50 (3.49-11.74)	0.950	1,182.17 (431.61−2,984.27)	4.97 (2.64-11.24)	0.971	0.003	0.952
**Lymphoma**	45	600 (365−1,015)	2.68 (1.47-4.78)	0.955	455.99 (267.14-900.29)	2.10 (1.10-4.69)	0.964	0.067	0.846
**Acute lymphoblast leukemia**	2	855 (540–855)^a^	3.37 (2.05–3.37)^a^	N/A	1,093.66 (632.88−1,093.66)^a^	4.31 (2.40–4.31)^a^	N/A	N/A	N/A
**G-CSF + plerixafor**									
**All**	28	753 (500−1,520)	3.44 (2.01-6.49)	0.953	575.46 (323.52−1,480.34)	2.76 (1.38-6.22)	0.965	0.017	0.912
**Multiple myeloma**	13	780 (637−2,175)	3.60 (2.24-9.62)	0.952	778.84 (473.56−1,889.53)	2.89 (1.72-7.99)	0.978	0.279	0.867
**Lymphoma**	15	690 (300−1,120)	3.27 (1.57-4.80)	0.946	543.99 (60.45-950.20)	2.20 (0.32-3.89)	0.971	0.017	0.946
**G-CSF**									
**Healthy donor**	29	1,580 (1,060−2,287)	9.18 (4.79-12.69)	0.951	1,430.45 (855.50−1,961.35)	8.10 (4.40-10.18)	0.955	0.005	0.905

Abbreviations: G-CSF, granulocyte colony-stimulating factor; HPC, hematopoietic stem cell; IQR, interquartile range; N/A, not applicable; PBSC, peripheral blood stem cell; Sysmex XN-HPC, Sysmex XN-derived hematopoietic progenitor cell enumeration

^a^Q3 not applicable for n = 2.

^b^*p*-values were obtained using the Wilcoxon signed-rank test to compare HPC and CD34 + derived cell yields.

## Discussion

The feasibility of stem cell transplantation is determined by the CD34 + cell count per kilogram of body weight. The timing of stem cell harvesting is also based on the CD34 + cell count in peripheral blood. The mobilization of PBSCs and the timing of apheresis are critical for efficiently collecting an adequate cell dose.

Estimating CD34 + cells in mobilized peripheral blood by flow cytometry on the morning of collection is considered the best predictor of a sufficient PBSC harvest. However, this method has limitations. It requires a long turnaround time, relies on costly antibody kits, and requires at least 1.5 hours of processing, even with experienced laboratory personnel. As a result, a simpler and faster method is needed to determine the optimal time of harvest.

HPC count enumeration using the XN-stem cell mode on the XN hematology analyzer has emerged as an alternative method. This method is cost-effective, requires no specialized expertise, and provides results with a rapid turnaround time of approximately 5 minutes.

Our study assessed the correlation between absolute HPC counts from the Sysmex XN-HPC and absolute CD34 + cell counts measured by flow cytometry. We observed a very strong correlation in both the pre-apheresis peripheral blood and PBSC apheresis groups (r = 0.917 and 0.932, respectively), including subgroup analyses. These findings align with prior research [13,15–18], which also showed strong correlations between XN-HPC and CD34 + flow cytometry in both peripheral blood and harvested PBSC products.

Despite this correlation, the median XN-HPC count was higher than the absolute CD34 + cell count, especially in MM patients. This pattern echoes reports by Dima et al. and Kim et al. [8,10]. One possible explanation is the presence of MM stem cells or myeloma-initiating cells, which exhibit tumor-initiating capacity, self-renewal, and resistance to chemotherapy [19,20]. Additionally, CD34‒ cells mobilized by the regimen may be indistinguishable from CD34 + cells during XN-HPC analysis. Certain blood cell precursors, including some CD34‒ cells [21–24], can appear in the same HPC detection region of the Sysmex XN-HPC [25]. These are not identified as CD34+ by flow cytometry and could account for discrepancies between HPC and flow cytometry results.

Prior research has reported various HPC cutoffs for achieving 2 × 10^6^ cells/kg of stem cell collection: Furundarena et al. [26] observed 35 cells/μL (sensitivity 78.6%, specificity 83.3%), Peerschke et al. [18] suggested 38 cells/μL, Tanosaki et al. [27] proposed 25 cells/μL or 56 cells/μL for safer collection, Gromme et al. [15] identified 19 cells/μL, and Kumar et al. [16] used 25 cells/μL (NPV 100%, PPV 66%). Importantly, our study focuses on establishing disease-specific HPC cutoff values directly targeting the transplant-critical endpoint of 2 × 10⁶ cells/kg. While previous studies [15,16,18,26,27] have reported various HPC thresholds, none have systematically derived cutoffs specifically for this clinically relevant transplant dose across different hematological malignancies. The disease-specific approach represents a significant advancement over the universal cutoff methodology employed in earlier research. Our finding that multiple myeloma requires a higher threshold (27 cells/μL) compared to lymphoma (20 cells/μL) challenges the individualized approach and introduces a precision medicine framework to PBSC collection that has not been previously established.

Variations among these cutoffs likely result from multiple factors. These include differences in study populations (e.g., disease type or ethnicity) and mobilization regimens (e.g., G-CSF, G-CSF plus chemotherapy, or G-CSF plus plerixafor). They may also reflect different Sysmex XN software versions, instrument settings, and calibration protocols. Such variability can influence HPC counts and lead to divergent cutoff values across studies.

Our findings show that the absolute HPC count accurately predicts CD34 + cell yield and strongly correlates with the absolute CD34 + cell count across all mobilization regimens. These correlations were not significantly affected by plerixafor mobilization, aligning with results from Villa et al. [28].

In the PBSC apheresis group, some cases displayed elevated HPC values, which could lead to an overestimation of CD34 + cells per kilogram of recipient body weight. However, the actual harvested CD34 + cell number may not match this predicted count. Interpreting results in PBSC apheresis therefore requires caution. Additionally, reagent quality is crucial. Expired Lysercell WDF, for instance, may fail to disrupt cell membranes effectively, potentially causing abnormally high HPC values.

The strengths of this study are multifaceted. First, we demonstrate that Sysmex XN-HPC serves as a rapid and cost-effective alternative to flow cytometry for optimizing PBSC collection timing, with strong correlation data validated across diverse hematological malignancies. Most importantly, our establishment of disease-specific cutoff values represents a significant advancement beyond simple threshold determination. By recognizing that lymphoma and multiple myeloma exhibit distinct mobilization characteristics requiring different HPC thresholds, clinicians can now implement precision-guided collection timing strategies. This represents a paradigm shift from the traditional one-size-fits-all methodology to a personalized medicine approach that accounts for disease-specific stem cell biology. Such precision has the potential to significantly impact healthcare economics by reducing unnecessary apheresis procedures while optimizing collection success rates. Our comprehensive analysis of both pre-apheresis and apheresis product samples further underscores the clinical relevance of these findings across the entire transplantation process.

However, the study has some limitations. First, smaller sample sizes in certain subgroups (particularly acute lymphoblastic leukemia with n = 2) restrict the generalizability of our results to all hematological malignancies. Second, potential confounders include heterogeneous mobilization regimens and variations in sample collection timing relative to G-CSF administration. A prospective, multicenter study validating these disease-specific cutoffs would enhance their bedside applicability and potentially guide more uniform PBSC collection guidelines.

In conclusion, this study establishes the first disease-specific HPC cutoff values directly targeting the clinically critical 2 × 10^6^ cells/kg threshold. The distinct cutoffs for multiple myeloma (27 cells/µL) versus lymphoma (20 cells/µL) represent a paradigm shift from universal threshold approaches to precision-guided PBSC collection, enabling optimized apheresis scheduling and potentially reducing healthcare costs. This advancement addresses a critical gap in current clinical practice by incorporating disease-specific stem cell biology into collection timing decisions.

## Supporting information

S1 Dataset Minimal dataset for this study, containing laboratory parameters for pre-apheresis and post-apheresis groups in separate sheets. (XLS)
